# Investigation of Displacement and Force Characteristics of Piezoelectric Multilayer Actuator for Active Preload Control System in Ultrasonic Motors

**DOI:** 10.3390/mi16121324

**Published:** 2025-11-26

**Authors:** Harsimran Singh Saini, Kristina Kilikevičienė, Andrius Čeponis

**Affiliations:** Institute of Mechanical Science, Faculty of Mechanics, Vilnius Gediminas Technical University, 10223 Vilnius, Lithuania

**Keywords:** piezoelectric ceramics, piezoelectric actuators, active preload control, ultrasonic piezoelectric motor

## Abstract

The paper represents both numerical simulations and experimental investigations of a piezoelectric active preload system that is foreseen to be applied to the active preload control system of rotors of ultrasonic piezoelectric motors. The investigated preload system is based on the piezoelectric multilayer actuator and disc-shaped spring, which is attached at end of the actuator. The total volume and mass of the preload system are 275 mm^3^ and 4.3 g, respectively. The results of investigations demonstrate strong agreement between the experimental and simulation data, showing nearly linear displacement and output force responses within an input voltage range of 20 V to 75 V in the frequency range from DC to 200 Hz. In the investigated ranges, the active preload system is able to ensure up to 1 N force with displacement amplitude up to 30 µm, which were obtained at a driving signal of 75 V. These results show that the investigated active preload system can replace passive preload devices in ultrasonic piezoelectric motors which are subjected to strict requirements in terms of their mass and mounting volume.

## 1. Introduction

Piezoelectric actuators and motors are well known for their high precision, low electromagnetic interference, minimal power consumption, and self-locking ability [1,2,3]. Unlike conventional systems, piezoelectric actuators and motors require low power, have low mass, are able to directly integrate into systems, lack the need for gear or reduction systems, can directly drive load, are noiseless, and are vacuum-compatible, making them especially ideal for small satellites or low-mass and -volume systems [4,5,6,7]. In addition, piezoelectric actuators and motors can be designed to handle loads with several degrees of freedom and without any changes in their structural design, as well as to produce linear and angular motions [8,9]. Lately, the latest advances in piezoelectric technology have led to the innovation of rotary piezoelectric motors, which provide precise motion crucial to accurate alignment necessary in free-space optical (FSO) communications or in other use cases where high precision motion is necessary [10,11,12].

Li et al. investigated the influence of different preloads of inertial piezoelectric actuators on how a preload force affects the output performance of the piezoelectric inertial actuator [13]. Their study presented a detailed structural model and an operating principle that correlates the preload force with the displacement of the actuator and the performance of the output supported by the experimental results. On the basis of the results, scholars have claimed that inadequate preload leads to undesired motion and decreases efficiency, whereas optimal preload achieves a balance between high output motion and speed. Their results concluded that an optimal preload is necessary to maintain repeatability and avoid excessive wear, which is crucial for aerospace applications where actuator reliability and accuracy are a necessity.

Hu et al. developed a variable pressure/position preload system for the spindle-bearing system by using piezoelectric actuators under closed-loop control [14]. This system had a PI controller to regulate the preload force based on feedback from force sensors, achieving dynamic compensation for thermal expansion, wear, and other operational disturbances. Their closed-loop strategy improved stiffness and vibration suppression in the spindle system while maintaining optimal preload throughout the operating cycle. Importantly, they demonstrated that piezoelectric-based active control could prevent preload degradation and enhance system longevity compared to traditional passive spring-based systems. This approach is highly relevant to precision motion systems such as piezoelectric motors, where preload stability directly affects positioning accuracy and operational repeatability.

Chen and Dwang reported on a ball screw drive mechanism with a piezoelectric nut for active ball screw preload and fine motion control [15]. Their design consists of high-speed coarse positioning and ultra-fine positioning. The system was used in combination, first to coarsely position at high speed with a light ball screw preload and then ultrafine positioning using the piezo electric nut at high preload. With this, they proved that the preload can be controlled electrically by variably exciting piezoelectric actuators. The experimental results of the system have shown that this mechanism could suppress backlash, minimize positioning errors, and improve stiffness in high-precision machining applications. The piezoelectric nut and preload made it possible to produce high resolution, high speed, and positioning accuracy up to a nanometer scale. Integrating the electromechanical preload system instead of the passive mechanical preload system not only enhanced static and dynamic stiffness but also enabled adaptive response by varying preload. These findings strongly demonstrate the importance of actively controlled preload systems for improving precision and robustness in rotary and linear motion applications.

Therefore, on the basis of the state of the art, it can be stated that active preload systems using piezoelectric actuators stand out among conventional passive preload systems and are able to ensure high motion resolution. Actively controlled preload systems outperform passive systems by maintaining the appropriate contact force under varying operating conditions, effectively prolonging the life of the motor by reducing wear and improving output performance [16]. In addition, during the design process of piezoelectric motors, they can be adapted to various designs of novel and complex systems to reduce the complexity of the system to overcome the typical drawbacks of conventional systems due to the fast response and low weight of piezoelectric materials [17]. Moreover, for piezoelectric rotary motors, where tribological contact between the stator and the rotor is essential, an active rotor preload control system offers an appealing way to compensate for wear-induced preload loss and temperature variations.

This study presents the design and analysis of an active preload control system intended for application in small-sized ultrasonic piezoelectric motors. The proposed preload mechanism, which integrates a piezoelectric multilayer actuator with a disc-shaped spring, offers a compact and lightweight solution for small-sized ultrasonic piezoelectric motors which are subjected to strict mass and mounting volume requirements. Compared to the state of the art, in most cases, preload systems are based on elliptical- or rhomb-type amplifiers, which adds additional mass and increased demand on mounting volume, while the proposed design excludes usage of these constructions via the integration of a disc-shaped spring. Therefore, owing to its small size, low mass, and reliable electromechanical performance, the developed active preload solution is particularly well-suited for integration into small-sized piezoelectric ultrasonic motors where minimal weight and compact dimensions are critical design requirements.

## 2. Design and Operation Principle of the Active Preload System

**Figure 1:**
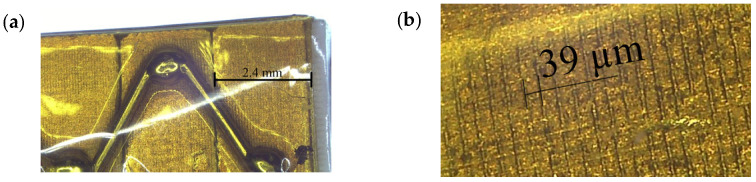
Internal composition of Thorlabs PK2FMP2 piezoelectric actuator: (**a**) multiple stacks, (**b**) magnified view of individual layers of a stack.

**Figure 2:**
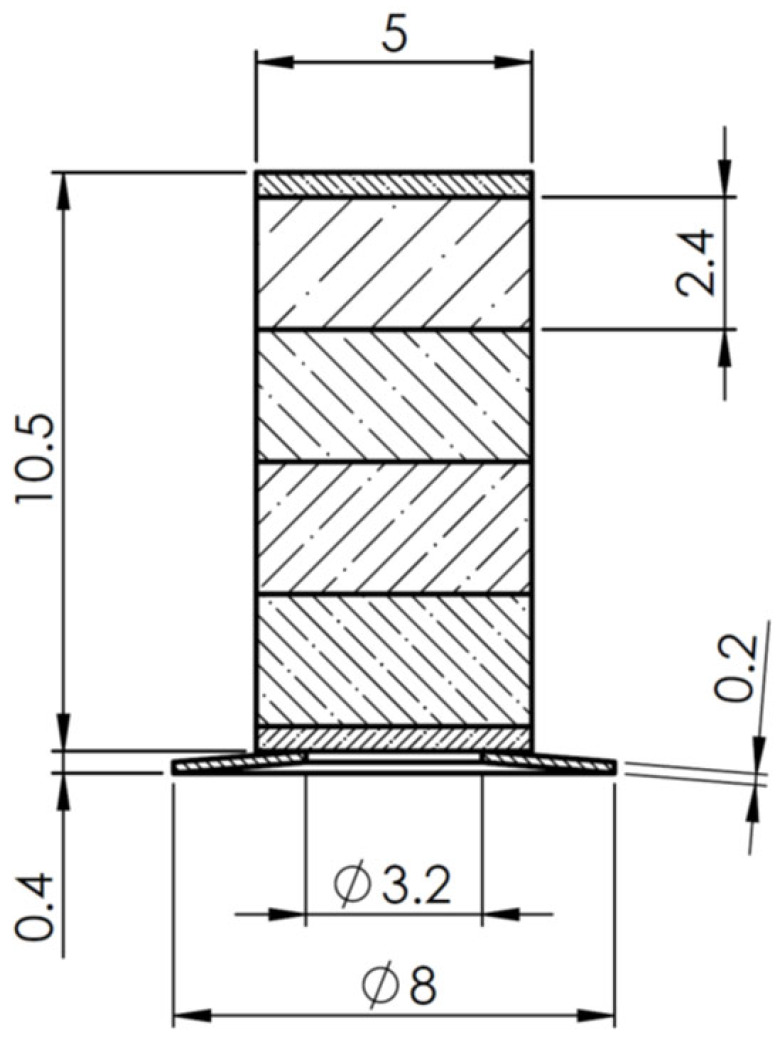
Section view of the active preload system.

The active preload system was based on the Thorlabs PK2FMP2 multilayer stack actuator (Thorlabs, Inc., Newton, NJ, USA) and a ring-shaped spring. The actuator has 1000 N blocking force and 11.2 µm free displacement amplitude, while the total height of the actuator is 10.5 mm with a square cross-section of 5 × 5 mm. The actuator comprises four individual stacks, each consisting of 61 layers of piezoelectric material THP51, while the ring-shaped spring was made of stainless steel 304 with a spring constant of 166.67 N/mm. The internal composition of the piezoelectric multilayer actuator and the design of the preload system are given in Figure 1 and Figure 2, respectively.

Therefore, the composition of the preload system was based on the piezoelectric multilayer actuator and ring-based spring that was placed at the free end of the actuator. The application of the active preload system is foreseen as follows: the disc-shaped spring is placed on the rotor via friction force while the free end of the actuator is preloaded by initial preload force, which would be based on the particular design of the motor. The operation principle of the preload system is based on longitudinal contraction and expansion of the piezoelectric multilayer that controls the preload of the ring-shaped spring. Furthermore, then the DC signal is applied to the preload system, the constant preload force, which is directly related to the displacement of the preload system, in tribological pair, is created and supported. In general, the preload system driven by the DC signal is equivalent to a spring, for which contraction and extension can be controlled actively. Such an operation of the preload system is suitable for piezoelectric ultrasonic motors, whose operation is based on harmonic vibrations, for example, a travelling wave, among others. On the other hand, when the AC driving signal is applied to the preload system, the preload force changes dynamically according to the amplitude and frequency of the driving signal. Such an operation principle of the preload system is primarily focused for application in ultrasonic piezoelectric motors, which operation is based on non-harmonic vibrations, for example, inertial motors, among others. In the first case, with the DC driving signal, the preload force can be changed in accordance with the changes in motor performance, which arises due to wear in tribological pair i.e., compensate the wear impact to motor dynamic characteristics. On the other hand, with an AC driving signal, the preload system is able to provide the possibility to not only compensate wear in the tribological pair but also dynamically reduce or remove backward motion in motors, whose operation is based on the principle of inertial movement through the dynamic change in the preload force during the slip stage.

Moreover, longitudinal displacement of the actuator can be continuously adjusted by varying the applied DC or AC voltage; the system enables precise control of preload force over a wide range of intermediate values, not just the maximum or minimum. In other words, different fractions of DC or AC voltage correspond to different levels of expansion/contraction, making it possible to obtain numerous output force levels. Additionally, the preload force range can be tailored not only by the applied voltage but also by changing the spring design (e.g., stiffness, geometry, or material of the disc spring). This provides flexibility to expand the achievable output force range and adapt the system to different applications.

## 3. Numerical Investigation of the Active Preload System

**Figure 3:**
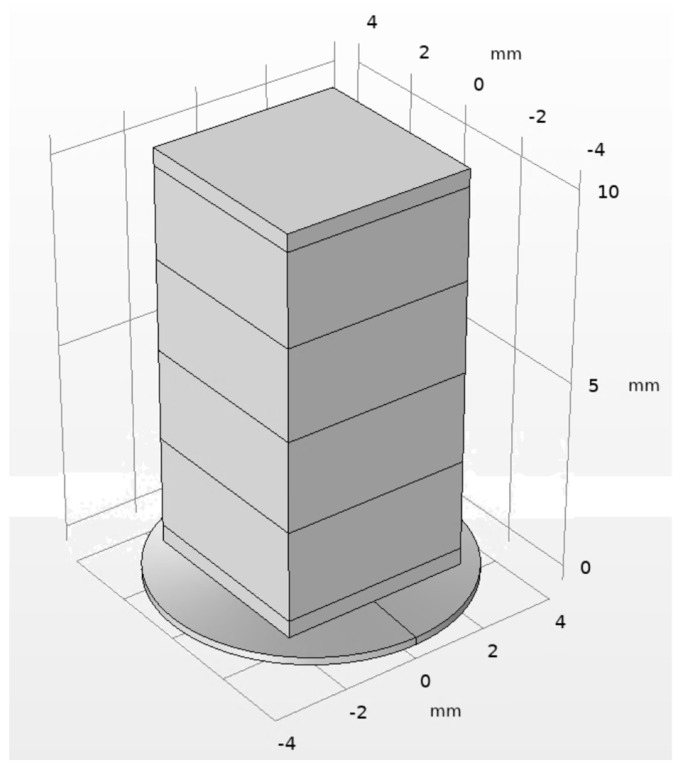
Numerical model of the active preload system.

Numerical calculations of an active preload system were carried out using COMSOL Multiphysics 6.3. The purpose of the investigations was to analyze the electromechanical behavior of the active preload system (Figure 2). Therefore, the numerical model (Figure 3) contained the following boundary conditions: the free end of the preload system was rigidly fixed, while the outer edge of the ring-shaped spring was defined as a sliding boundary condition in order to simulate contact with the motor rotor. The material characteristics were also included in the model; that is, the layers of piezoelectric material were defined as being made of THP51, while the top and bottom passive layers of the multilayer were defined as being made of alumina oxide. Finally, a disc-shaped spring was defined as being made of 304 stainless steel. The material characteristics used to build the numerical model are given in Table 1.

**Figure 4:**
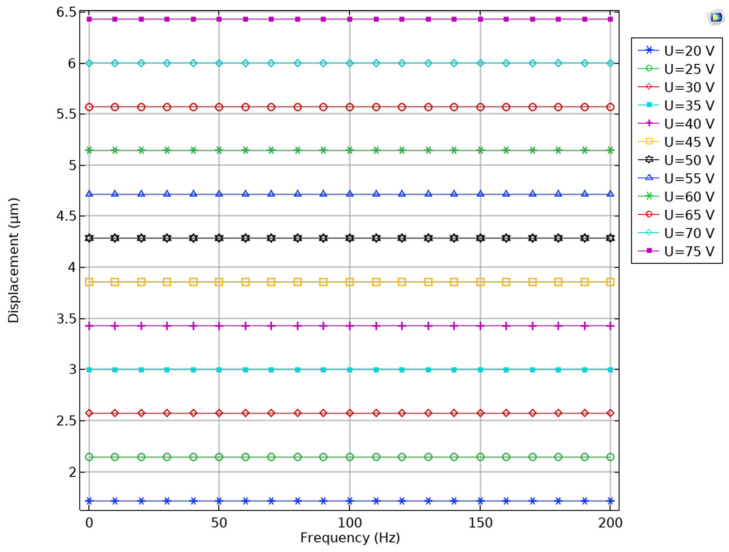
Displacement–frequency characteristics of the active preload system.

The first stage of the numerical investigations was dedicated to the calculation of the displacement amplitudes of the active preload system, while different amplitudes of the DC and AC signals are applied with different frequencies. The frequency range was set from DC to 200 Hz. The DC signal, as it was stated before, is relevant for piezoelectric motors, for which the operation is based on harmonic excitation, while a frequency range up to 200 Hz ensures that the preload system will be able to manage angular motion speeds of rotor up to 12,000 RPM and ensure reduction or removal of backward motion of rotor, while the preload system is used in dynamic preload control mode. In addition, the amplitudes of the excitation signal were set to range from 20 V_p-p_ to 75 V_p-p_ with an increment step of 5 V_p-p_. The results of the calculations are shown in Figure 4.

During analysis of the results, it can be found that the displacement–voltage–frequency characteristics have linear dependency. In other words, considering the voltage amplitude, the potential displacement amplitude of the preload system in the frequency range from DC to 200 Hz can be directly calculated. Therefore, the lowest displacement amplitude was obtained while the driving signal amplitude was set to 20 V_p-p_ and reached 1.72 µm or 86.1 nm/V_p-p_. On the other hand, the highest displacement amplitude was obtained while the driving signal was set to 75 V_p-p_ and reached 6.4 or 85.3 nm/V_p-p_. Therefore, it can be noted that despite the frequency and amplitude of the excitation signal, the preload system has a linear and predictable response to the driving signal, which ensures simple driving and control of the system.

**Figure 5:**
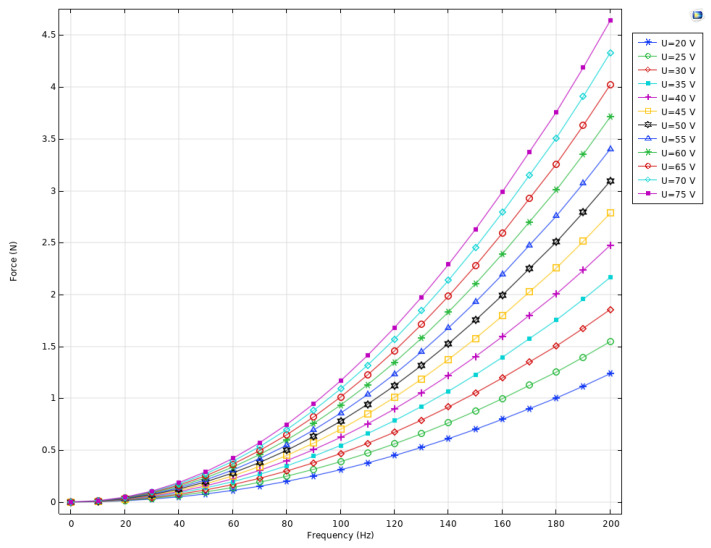
Output force–frequency characteristics of active preload system.

The next stage of the numerical investigation was dedicated to the calculation of output force–driving signal amplitude–frequency characteristics of the active preload system. The boundary conditions of the model as well as the frequency ranges and driving signal amplitudes were set as in the previous case. The results of the calculations are shown in Figure 5.

The output force analysis (Figure 5) revealed an almost linear relationship with the applied voltage, without noticeable resonance peaks throughout the entire investigated frequency spectrum. This indicates that the system operates within its linear regime and maintains dynamic stability under excitation, which is essential for reliable preload control in ultrasonic piezoelectric motors. The observed change in output force with frequency is attributed to the dynamic interaction between the piezoelectric multilayer actuator and the elastic disc-shaped spring. As the excitation frequency rises, the actuator undergoes more rapid strain cycles, resulting in higher acceleration of the attached spring mass and, consequently, a larger dynamic reaction force.

Overall, the numerical results confirm that the proposed preload system—based on a multilayer piezoelectric actuator and a disc-shaped spring—ensures both high linearity and dynamic stability while maintaining a compact and lightweight design. These characteristics make it particularly well-suited for integration into small-sized piezoelectric ultrasonic motors, where precise, stable, and space-efficient preload control is required.

## 4. Experimental Investigation of the Active Preload System

**Figure 6:**
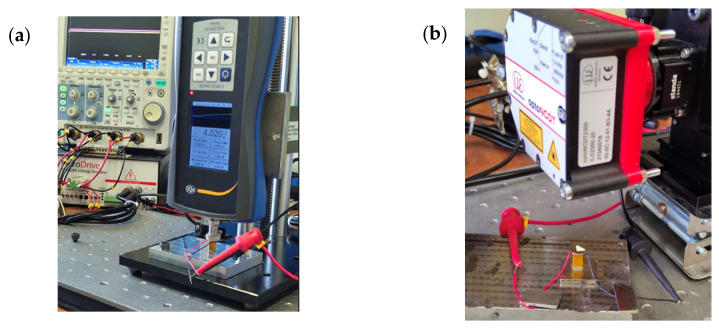
The prototype of the preload system and experimental setup; (**a**) setup for output force measurements; (**b**) setup for displacement measurements.

Experimental investigations were performed with the goal of indicating electromechanical characteristics of the preload system. The prototype of the preload system was made for this purpose, in accordance with Figure 2. Also, an experimental setup was built in order to perform the investigations. A view of the prototype incorporated into the experimental setup is given in Figure 6.

**Figure 7:**
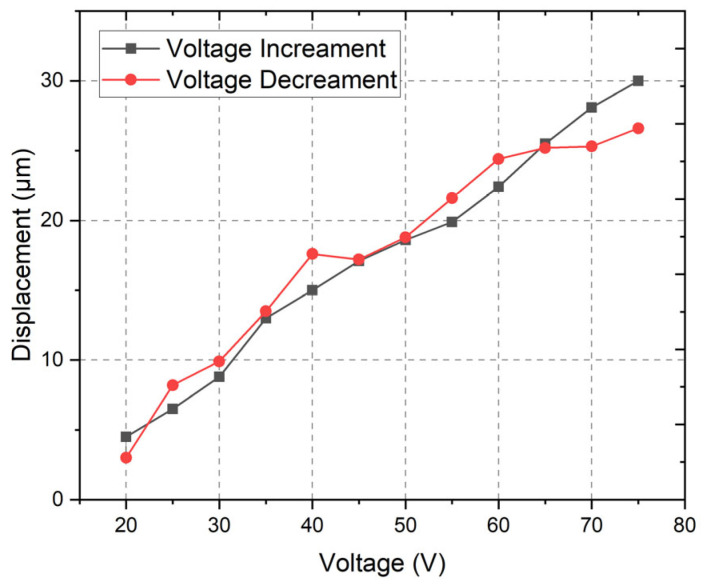
Displacement–voltage amplitude characteristics of the preload system.

Therefore, the first stage of the experimental investigations was dedicated to the measurement of displacement amplitudes under different excitation signal amplitudes, i.e., from 20 V_DC_ to 75 V_DC_ with an increment step of 5 V_DC_. The excitation signal was generated by the Tabor Electronics WW5064 signal generator (Tabor Electronics Ltd., Nesher, Israel), while the signal was amplified by the Piezo Drive signal amplifier PX200X4 (PiezoDrive Pty. Ltd., Newcastle, NSW, Australia). The signal amplitude was controlled by a Yokogawa DLM 2024 oscilloscope (Yokogawa Test & Measurement, Tokyo, Japan). The displacement amplitudes of the preload system were measured using the Micro Epsilon ILD 2300-20 displacement sensor (Micro-Epsilon Messtechnik GmbH & Co. KG, Ortenburg, Germany). The results of the measurements are shown in Figure 7.

Figure 7 shows a generally linear trend across the tested voltage range, with only minor deviations between voltage increment and decrement cycles. The minimum displacement is observed at 20 V_DC_, yielding values close to 3–4 μm, while the maximum displacement reaches about 30 μm at 75 V_DC_. The difference between increment and decrement voltage graphs mainly occurs due to the effect of hysteresis, as well as possible measurement errors. However, the characteristics show that the actuator provides a wide displacement window suitable for controlled preload adjustment.

The near-linear relationship between voltage and displacement indicates that the system operates within its intended linear regime, ensuring predictable actuation performance. The slight hysteresis observed between the voltage increment and decrement curves reflects the typical behavior of a spring-based system. However, it remains relatively small, suggesting stable repeatability. Overall, the results confirm that the active preload system exhibits both sufficient displacement amplitude and a near-linear response, making it well-suited for applications requiring precise and controllable preload forces.

**Figure 8:**
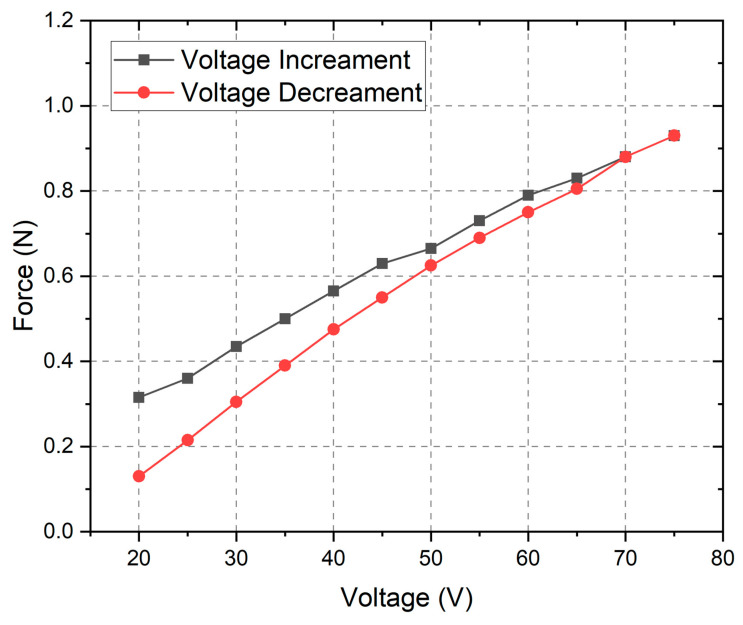
Output force–voltage amplitude characteristics of the preload system.

The final stage of the experimental investigation was dedicated to the measurement of the output force of the preload system, while different amplitudes of the excitation signal were used to drive the system. The driving signal amplitudes, generation, amplification, and control were implemented as in the case before, while the output force was measured by the PCE DFG N force sensor (PCE Deutschland GmbH, Meschede, Germany) placed on top of the preload system (Figure 6b). The sensors have a 10 N maximum measuring range with a measuring resolution of 5 mN. The results of the measurements are given in Figure 8.

As can be found in Figure 8, the output force is dependent on the driving signal amplitude, while the amplitudes increase and decrease. The system produces its lowest force at 20 V_DC_, where values are between 0.1 N and 0.2 N, and its highest force at 75 V_DC_, reaching close to 1.0 N. Across this range, the relationship between voltage and force is proportional, demonstrating a nearly linear response.

A modest hysteresis can be indicated between the two voltage cycles: the decrement curve consistently yields slightly lower forces than the increment curve, especially at lower voltages. Nevertheless, the overall trend remains stable and predictable. These characteristics suggest that the preload system can deliver a controlled and repeatable force output while maintaining functional linearity despite minor hysteretic effects.

On the other hand, it must be noticed that temperature variations can significantly affect the hysteresis behavior and output force characteristics of the preload system. As temperature increases, the dielectric and piezoelectric coefficients of the ceramic material, i.e., THP51, typically change, leading to altered polarization dynamics and a shift in the actuator’s strain–electric field relationship. Elevated temperatures may increase internal domain mobility, which can initially reduce hysteresis, while at lower temperatures, reduced domain wall movement increases the effect of hysteresis. Such behavior can limit the actuator’s displacement and force linearity, especially in environments with dynamic temperatures. Therefore, it should be considered during the design stage and application requirements; the definition and compensation of this affect must be included to the control algorithm of the preload system.

Overall, the experimental findings provide good validation of the numerical model, confirming both the accuracy of the predictive framework and the reliability of the performance of the preload system under the tested conditions. The system demonstrates the ability to deliver stable, predictable, and tunable forces across the specified voltage range, highlighting its effectiveness for precision control applications. Importantly, the nearly linear voltage–force and voltage–displacement relationships, combined with the absence of significant dynamic instabilities, underscore the suitability of the system to use the system with ultrasonic motors.

## 5. Conclusions

This study presented the design, numerical modeling, and experimental validation of an active preload control system based on a multilayer piezoelectric actuator integrated with a disc-shaped spring for ultrasonic piezoelectric motors. The investigations confirmed that the proposed preload system exhibits a highly linear and predictable electromechanical response, with displacement and output force both showing near-linear dependence on the applied voltage across the range of 20 V–75 V and frequencies up to 200 Hz. The numerical simulations and experimental measurements demonstrated strong agreement, validating the accuracy of the developed model. Experimentally, the system achieved displacements of up to 30 µm and output forces of approximately 1 N, ensuring sufficient preload capability for small-scale ultrasonic motors. The preload system also maintained dynamic stability throughout the entire tested frequency range, with no observed resonance peaks, confirming its suitability for both static and dynamic preload control. A key advantage of the developed system is its compact and lightweight design—with a total volume of only 275 mm^3^ and a mass of 4.3 g—which allows for its straightforward integration into small-sized ultrasonic piezoelectric motors operating under strict spatial and weight constraints. Additionally, the system’s low hysteresis, excellent repeatability, and wide controllable range of displacement and force enable reliable long-term operation and make it adaptable for environments requiring high precision and stability.

## Figures and Tables

**Table 1:** Characteristics of the materials.

Material Properties	THP51	Alumina Oxide	S.S. 304
Density [kg/m^3^]	7700	3900	8000
Young’s modulus [N/m^2^]	5 × 10^10^	41.9 × 10^10^	19.3 × 10^10^
Poisson’s ratio	0.32	0.2	0.29
Isotropic structural loss factor	-	-	-
Relative dielectric constant	Ɛ^T^_33_/Ɛ_0_ = 3300	-	-
Elastic compliance coefficient [10^−12^ m^2^/N]	S_11_^E^ = 17, S_13_^E^ = 23	-	-
Elastic stiffness coefficient c_33_^D^ [N/m^2^]	11 × 10^10^	-	-
Piezoelectric constant d^33^ [10^−12^ m/V]	710	-	-
Piezoelectric constant d^31^ [10^−12^ m/V]	−320	-	-
Coefficient of thermal expansion [1/K]	3.5 × 10^−6^	8 × 10^−6^	16 × 10^−6^
Thermal conductivity [W/(m·K)]	1.5	27	16.2

## Data Availability

The original contributions presented in the study are included in the article; further inquiries can be directed to the corresponding author.

## References

[1] Katzir S. (2003). The Discovery of the Piezoelectric Effect. Arch. Hist. Exact Sci..

[2] Dong S. (2012). Review on piezoelectric, ultrasonic, and magnetoelectric actuators. J. Adv. Dielectr..

[3] Mohith S., Upadhya A.R., Navin K.P., Kulkarni S.M., Rao M. (2021). Recent trends in piezoelectric actuators for precision motion and their applications: A review. Smart Mater. Struct..

[4] Chi Z., Xu Q. (2014). Recent advances in the control of piezoelectric actuators. Int. J. Adv. Robot. Syst..

[5] DongMei X., BingJie Z., SiMiao Y., XuHui Z. (2023). Review on Single-Phase Driven Ultrasonic Motors. J. Intell. Mater. Syst. Struct..

[6] Morita T., Takahashi S., Asama H., Niino T. (2003). Rotational feedthrough using an ultrasonic motor and its performance in ultra high vacuum conditions. Vacuum.

[7] Delibas B., Koc B. (2020). A Method to Realize Low Velocity Movability and Eliminate Friction Induced Noise in Piezoelectric Ultrasonic Motors. IEEE/ASME Trans. Mechatron..

[8] Chang Q., Chen W., Zhang S., Deng J., Liu Y. (2024). Review on Multiple-Degree-of-Freedom Cross-Scale Piezoelectric Actuation Technology. Adv. Intell. Syst..

[9] Zhou X., Wu S., Wang X., Wang Z., Zhu Q., Sun J., Huang P., Wang X., Huang W., Lu Q. (2024). Review on piezoelectric actuators: Materials, classifications, applications, and recent trends. Front. Mech. Eng..

[10] Milaševičius M., Mačiulis L. (2024). A Review of Mechanical Fine-Pointing Actuators for Free-Space Optical Communication. Aerospace.

[11] Muhire D., Stepanova D., Santra S., Baranwal P., Romero M., Amrutkar R., Bonnart S., Jha D., Zucherman A. Optical Communications for Small Satellites: A Review of Pointing Strategies & Requirements Optimization. Proceedings of the International Astronautical Congress (IAC).

[12] Grenfell P., Aguilar A., Cahoy K., Long M. Pointing, Acquisition, and Tracking for Small Satellite Laser Communications. Proceedings of the 32nd Annual AIAA/USU Conference on Small Satellites.

[13] Li Y., Yuan L., Wang L. Research on Preload Characteristics of the Inertial Piezoelectric Actuator. Proceedings of the 2023 17th Symposium on Piezoelectricity, Acoustic Waves, and Device Applications (SPAWDA).

[14] Hu G., Zhang D., Gao W., Chen Y., Liu T., Tian Y. (2018). Study on Variable Pressure/Position Preload Spindle-Bearing System by Using Piezoelectric Actuators Under Close-Loop Control. Int. J. Mach. Tools Manuf..

[15] Chen J.S., Dwang I.C. (2000). A Ballscrew Drive Mechanism with Piezo-Electric Nut for Preload and Motion Control. Int. J. Mach. Tools Manuf..

[16] Li Z., Zhao H., Che S., Chen X., Sun H. (2022). Analysis of Preload of Three-Stator Ultrasonic Motor. Micromachines.

[17] Tamburrano P., Sciatti F., Plummer A.R., Distaso E., De Palma P., Amirante R. (2021). A Review of Novel Architectures of Servovalves Driven by Piezoelectric Actuators. Energies.

